# Metabolic Interaction of the Active Constituents of *Coptis chinensis* in Human Liver Microsomes

**DOI:** 10.1155/2015/802903

**Published:** 2015-01-06

**Authors:** Songcan Liu, Xinfeng Zhang, Furong Qiu, Ping Miao, Shujiao Shen, Leilei Zhu, Jin Zeng, Jian Jiang

**Affiliations:** Department of Clinical Pharmacology, Shuguang Hospital Affiliated to Shanghai University of Traditional Chinese Medicine, Shanghai 201203, China

## Abstract

*Coptis chinensis* is commonly used in traditional Chinese medicine. The study investigated metabolic interaction of the active constituents (berberine, coptisine, palmatine, and jatrorrhizine) of *Coptis chinensis* in human liver microsomes. After incubation of the four constituents of *Coptis chinensis* in HLMs, the metabolism of the four constituents was observed by HPLC. The *in vitro* inhibition experiment between the active constituents was conducted, and IC_50_ value was estimated. Coptisine exhibited inhibitions against the formation of the two metabolites of berberine with IC_50_ values of 6.5 and 8.3 *μ*M, respectively. Palmatine and jatrorrhizine showed the weaker inhibitory effect on the formation of the metabolites of berberine. Berberine showed a weak inhibitory effect on the production of coptisine metabolite with an IC_50_ value of 115 *μ*M, and palmatine and jatrorrhizine had little inhibitory effect on the formation of coptisine metabolite. Berberine, coptisine, and jatrorrhizine showed no inhibitory effect on the generation of palmatine metabolite (IC_50_ > 200 *μ*M). The findings suggested that there are different degrees of metabolic interaction between the four components. Coptisine showed the strongest inhibition toward berberine metabolism.

## 1. Introduction

Huanglian (*Coptis chinensis*), the rhizome of* Coptis chinensis* Franch from the Ranunculaceae family [1], has been used for hundreds of years in China and other oriental countries. The major active constituents of* Coptis chinensis* are isoquinoline alkaloids, including berberine, coptisine, palmatine, and jatrorrhizine [2]. The isoquinoline alkaloids are responsible for its various pharmacological effects, such as antibacterial [3], blood glucose-lowering [4] and lipid-lowering [5] effects.* Coptis chinensis* is widely used either alone or in combination with other herbs for patients with gastroenteritis, diabetes, and hyperlipidemia.

Some reported that berberine was metabolized mainly by CYP2D6 in HLMs [6, 7]. The metabolites of jatrorrhizine [8] have been analyzed in liver microsomes of rat. Demethylation of jatrorrhizine has been shown to be catalyzed by CYP3A1/2 and CYP2D2 in RLMs [9]. Furthermore, the constituents of* Coptis chinensis* have also the ability to inhibit CYP activities [10]. Some studies suggested that the availability of berberine appeared extremely low after oral administration of berberine in human and rats [11, 12]. Our previous study suggested that the AUC and *C*_max⁡_ of berberine increased significantly in rats receiving* Coptis chinensis* extract comparing with those receiving the pure berberine (data not shown). So, it was assumed that the coexisting constituents in* Coptis chinensis* could enhance the oral absorption and bioavailability of berberine via metabolic interaction among these constituents of* Coptis chinensis*. However, metabolic interaction of the herbal constituents of Rhizoma Coptidis alkaloid in human liver microsomes has not been reported.

The objective of the present work was to investigate metabolic interaction of these active constituents (berberine, coptisine, palmatine, and jatrorrhizine) of* Coptis chinensis *in HLMs and to exploit metabolism-based mechanism of enhancing the oral absorption and bioavailability of the active constituents of* Coptis chinensis*.

## 2. Materials and Methods

### 2.1. Chemicals and Reagents

Berberine hydrochloride, coptisine hydrochloride, palmatine hydrochloride, and jatrorrhizine hydrochloride were purchased from the National Institute for the Control of Pharmaceutical and Biological Products (Beijing, China). *β*-Nicotinamide adenine dinucleotide phosphate reduced tetrasodium salt (NADPH) was purchased from Sigma-Aldrich Co. (St. Louis, MO, USA). HPLC-grade methanol and acetonitrile were obtained from Tedia Company Inc. (USA). Phosphate-buffered saline (PBS, 0.1 M) was supplied by Gibco Laboratories (MD, USA). Deionized water was purified using a Milli-Q system (Millipore Corporation, USA). Dimethyl sulfoxide (DMSO), ammonium acetate, and other chemicals were all of analytical grade and were supplied by Sinopharm Chemical Reagent Co. Ltd. (Beijing, China).

### 2.2. Preparation of Standard and Stock Solutions

Berberine, coptisine, palmatine, and jatrorrhizine were dissolved in DMSO. NADPH was dissolved in PBS. NADPH was prepared daily and kept on ice until use. The solution above was diluted 100 times with PBS before adding to the incubation mixture. The final DMSO, acetonitrile, and methanol concentration in the incubation mixture was 0.05% v/v.

### 2.3. Human Liver Microsomes

HLMs used in this study were provided by the Research Institute for Liver Diseases Co. Ltd. (Shanghai, China) and stored at −80°C until use. The microsomes were prepared from ten Mongolian individual human donor livers.

### 2.4. Incubation Procedure [13, 14]

A typical incubation mixture was prepared in a total volume of 200 *μ*L as follows: 40 *μ*L HLMs (1 mg/mL), 20 *μ*L NADPH (10 mM), 10 *μ*L substrate and/or 10 *μ*L inhibitor, and 130 or 120 *μ*L PBS (0.1 M, pH 7.4). There was a 5 min preincubation period at 37°C before the reaction was initiated by the addition of NADPH. The reaction then proceeded for 30 min at 37°C in a shaking water bath.

Controls without NADPH and without HLMs were performed to ensure that the formation of metabolites was dependent on HLMs and NADPH.

### 2.5. Enzyme Kinetics Analysis

Berberine, coptisine, or palmatine as the substrate (final concentrations ranging from 2.5 to 200 *μ*M) was incubated in the mixture with HLMs and NADPH at 37°C for 30 min. The *K*_*m*_ and *V*_max⁡_ values were determined by nonlinear regression analysis using the Michaelis-Menten equation: *V* = *V*_max⁡_ × [*S*]/(*K*_*m*_ + [*S*]), where *V*_max⁡_ is the maximal velocity of formation, [*S*] is the concentration of the substrate, and *K*_*m*_ is the substrate concentration at half-maximal velocity.

### 2.6. Interaction between One Constituent and Other Constituents of* Coptis chinensis* in HLMs

When one of the three constituents (berberine, coptisine, or palmatine) was used as a substrate, the other two constituents and jatrorrhizine were used as inhibitors. The final concentration of the constituent of* Coptis chinensis* as a substrate was 10 *μ*M, and the final concentration range of the* Coptis chinensis* constituents as inhibitors was from 0.5 to 200 *μ*M. These inhibitors and substrates were preincubated in the presence of HLMs at 37°C for 5 min. NADPH was then added and the reaction mixture was incubated another 30 min.

### 2.7. Sample Preparation and HPLC Analysis

The reactions were terminated by the addition of ice-cold acetonitrile (200 *μ*L), followed by vortexing for 3 min and centrifugation at 20,000 rpm for 10 min at 4°C to remove the denatured proteins. The supernatant (20 *μ*L) was injected into the HPLC (Agilent, Germany) system. An Agilent series 1200 HPLC system was equipped with degasser, quaternary pump, autosampler, and UV detector. Chromatographic separation was achieved on an Agilent Eclipse XDB-C18 (4.6 mm × 150 mm, 5 *μ*m) with mobile phase of 20 mM ammonium acetate and 0.1% formic acid in water (A)-methanol (B) at a flow rate of 1.0 mL/min. The gradient program was used as follows: 0–5 min, 20%B; 5–15 min, 20%B–35%B; 15–25 min, 35%B–45%B; and 25.1–30 min, 20%B. The column temperature was maintained at 40°C. The peaks were determined using a UV detector set at a wavelength of 354 nm.

### 2.8. Data Analysis

All results are expressed as the mean ± standard deviation (SD) of the estimates obtained from the three different HLMs experiments performed in triplicate. The relative amounts of berberine, palmatine, and coptisine metabolites were expressed as the peak area of the metabolites formed. The percent inhibition was calculated from the ratio of the amount of metabolites formed with and without the specific inhibitor, and the 50% inhibitory concentration (IC_50_) values and enzyme kinetic parameters *K*_*m*_ and *V*_max⁡_ were calculated using GraphPad Prism 5.04 (GraphPad Prism, Inc., San Diego, CA, USA). The intrinsic clearance (Cl_int⁡_) is evaluated according to CL_int⁡_ = *V*_max⁡_/*K*_*m*_.

## 3. Results

### 3.1. Identification of Metabolites of Berberine, Coptisine, and Palmatine with HLMs

**Figure 1:**
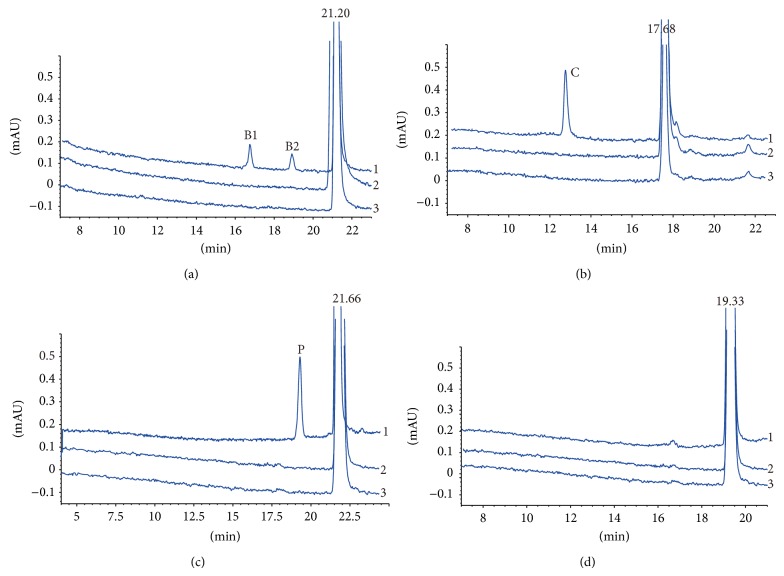
HPLC chromatograms of berberine, coptisine, palmatine, jatrorrhizine, and their metabolites in HLMs. Two metabolites (B1, B2) and berberine were eluted at 16.79, 18.94, and 21.20 min, respectively (a). Metabolite (C) and coptisine were eluted at 12.83 and 17.68 min, respectively (b). Metabolite (P) and palmatine were eluted at 21.66 and 19.3 min, respectively (c). Jatrorrhizine was eluted at 19.33 min (d). (1) Incubation with NADPH in HLMs, (2) no incubation with NADPH in HLMs, and (3) incubation with HLMs without NADPH.

When berberine, coptisine, palmatine, or jatrorrhizine was incubated with HLMs and NADPH for 30 min, two metabolites, one metabolite, and one metabolite of berberine, coptisine, and palmatine were, respectively, observed by HPLC, but no metabolite was observed for jatrorrhizine (Figure 1).

### 3.2. Enzymatic Kinetic Parameters for Berberine, Coptisine, and Palmatine Metabolites in HLMs

The *K*_*m*_ values for the metabolites of berberine, coptisine, and palmatine in the presence of HLMs were 32.24, 32.83, 36.35, and 87.47 *μ*M, respectively (Table 1). The *V*_max⁡_ values for the metabolites of berberine, coptisine, and palmatine in HLMs were 4.474, 3.371, 1.808, and 3.147 Area/min/mg protein, respectively (Table 1). The Cl_int⁡_ values for the metabolites of berberine, coptisine, and palmatine were 0.13, 0.10, 0.05, and 0.03 mAU/mg pro/*μ*M, respectively (Table 1).

### 3.3. Interaction between One Constituent and Other Constituents of* Coptis chinensis* in HLMs

In HLMs, coptisine decreased the formation of the two metabolites (B1 and B2) of berberine to a similar extent with IC_50_ values of 6.5 and 8.3 *μ*M, respectively. The generation of metabolites (B1 and B2) of berberine was slightly inhibited by palmatine with IC_50_ values of 185 and 78.5 *μ*M, respectively. The production of metabolites (B2) was inhibited by jatrorrhizine with an IC_50_ value of 28.5 *μ*M, whereas jatrorrhizine had little inhibitory effect on the formation of B1 (IC_50_ > 200 *μ*M) (Table 2).

Berberine showed an inhibitory effect on the production of coptisine metabolite with an IC_50_ value of 115 *μ*M. In addition, palmatine and jatrorrhizine had little inhibitory effect on the formation of coptisine metabolite (IC_50_ > 200 *μ*M) (Table 2).

In the presence of HLMs, berberine, coptisine, and jatrorrhizine showed no inhibitory effect on the generation of palmatine metabolite (IC_50_ > 200 *μ*M) (Table 2).

## 4. Discussion

This is investigation of metabolic interaction of the active constituents of* Coptis chinensis* (berberine, coptisine, palmatine, and jatrorrhizine) in human liver microsomes for the first time. In this study, two metabolites, one metabolite, and one metabolite of berberine, coptisine, and palmatine were observed by HPLC but no metabolite of jatrorrhizine was observed after incubation of the four constituents of* Coptis chinensis* in HLMs with NADPH. LC-MS/MS was used as a guide to identify these metabolites. B1 corresponded to an [M]^+^ ion at* m/z* 324, which was 12 Da less than that of berberine, suggesting that B1 was a demethylated ring-opened product of berberine. B2 had an [M]^+^ ion at* m/z* 322, which was a loss of 14 Da (CH_2_) compared with berberine, and the metabolite (C) of coptisine had an [M]^+^ ion at* m/z* 308, which was 14 Da (CH_2_) lower than that for coptisine, and the metabolite (P) of palmatine had an [M]^+^ ion at* m/z* 338, which was 14 Da (CH_2_) lower than that of palmatine. These findings were consistent with the results of some reports [15–17] and suggested that berberine, coptisine, and palmatine could produce certain amount of phase I metabolites in HLM via oxidative demethylation.

Using recombinant human CYP enzyme and chemical inhibition analysis in HLMs, we found that berberine, coptisine, and palmatine were metabolized by CYP2D6, CYP3A4, and CYP1A2. CYP2D6 was the predominant enzyme involved in the metabolism of berberine (consistent with Guo's finding [7]) and coptisine, while CYP1A2 was the primary contributor toward palmatine metabolism.

The enzymatic kinetic studies revealed that the* in vitro* intrinsic clearance (CL_int⁡_) values for the formation of two berberine metabolites in HLMs were approximately 2 to 3-fold higher than those of coptisine and palmatine.

In this study, we found that there were different degrees of metabolic interaction between the four components. Berberine showed a weak inhibitory effect on the production of coptisine metabolite with an IC_50_ value of 115 *μ*M. Palmatine and jatrorrhizine had little inhibitory effect on the formation of coptisine metabolite. Furthermore, berberine, coptisine, and jatrorrhizine showed no inhibitory effect on the generation of palmatine metabolite (IC_50_ > 200 *μ*M). However, coptisine showed the strongest inhibition toward berberine metabolism. As described above, berberine was metabolized mainly via CYP2D6 in HLMs and produced two major metabolites (B1 and B2), while coptisine had a strong inhibitory effect on CYP2D6 with IC_50_ values of 4.4 *μ*M [10]. Coptisine decreased the amount of B1 and B2 produced with IC_50_ values of 6.5 and 8.3 *μ*M in HLMs, respectively. It indicated that the production of B1 and B2 was decreased in liver due to CYP2D6 being inhibited by coptisine.

The alkaloids in* Coptis chinensis *are poorly absorbed when taken orally, resulting in low oral bioavailability [12, 15, 18, 19]; the plasmas concentration of coptisine cannot reach 6.5 *μ*M. However, this concentration can be reached in liver because of tissue distribution [11].

In conclusion, coexisting constituents (coptisine) in* Coptis chinensis* inhibited the metabolism of berberine in liver and might increase its bioavailability. The present finding provides novel insight into the understanding of the metabolism-based synergistic mechanism of the coexisting constituents in herb.

## Figures and Tables

**Table 1:** Enzymatic kinetic parameters for berberine, coptisine, and palmatine metabolites in HLMs.

Metabolites	*K*_*m*_ (*μ*M)	*V*_max⁡_ (Area/min/mg pro)	CL_int⁡_ (Area/min/mg/pro/*μ*M)
B1	32.24	4.174	0.13
B2	32.83	3.071	0.10
C	36.35	1.808	0.05
P	87.47	2.447	0.03

Note: B1, metabolite 1 of berberine; B2, metabolite 2 of berberine; C, metabolite of coptisine; P, metabolite of palmatine.

**Table 2:** The IC_50_ values for interaction between one constituent and other constituents of *Coptis chinensis *in HLMs (*μ*M).

Metabolites	Ber	COP	Pal	Jat
B1	—	6.5	185	>200
B2	—	8.3	78.5	28.5
C	115	—	>200	>200
P	>200	>200	—	>200
